# Down syndrome‐associated transient abnormal myelopoiesis with placental involvement

**DOI:** 10.1002/jha2.495

**Published:** 2022-09-20

**Authors:** Yahya Daneshbod, Ravi Raghavan

**Affiliations:** ^1^ Department of Pathology and Laboratory Medicine Loma Linda University Medical Center Loma Linda California USA

**Keywords:** down syndrome, GATA‐1, MYELOID LEUKAEMIA

1

A 2‐day‐old Down syndrome (DS) neonate born at 35 weeks 5 days presented with leukocytosis and blasts showing cytoplasmic blebs (Panel1×600). By flow‐cytometry, blasts were positive for CD34, CD61 and CD71 consistent with megakaryoblasts. Based on fetoplacental circulation (Figure), blasts lodged in the umbilical cord, chorionic plate, villi and membranes (Panels 2–5, H&E×100). Megakaryoblasts were generally confined to vascular lumina, but they infiltrated their walls (Panel 2 right, H&E×100). Immunohistochemically, blasts expressed CD61 (Panels 2,4,5×100 objective). Leukocytosis and increased blasts resolved within two weeks. These findings are consistent with DS‐associated transient abnormal myelopoiesis (TAM) with placental involvement. Somatic mutations of GATA1 are involved in the pathogenesis of DSTAM. GATA1 gene is necessary for the development of megakaryocytic lineages. Trisomy 21 perturbs fetal hematopoiesis, providing the context for: transformation of these fetal hematopoietic cells by acquired mutations in the GATA1 gene to produce DSTAM. Eighty percent of DSTAM cases resolve spontaneously within three months, coinciding with the transition of hematopoiesis from fetal liver to bone marrow. Fetal and maternal malignancies can cross and metastasize to the placenta. In fetal metastasis, tumor cells stay confined to the fetal vessels, while, in maternal malignancies, cells infiltrate the peri‐villous(maternal) space (Panel 6*). Patient's parental verbal and written consent was received.

Figure: Blasts showing cytoplasmic blebs (Panel1 × 600). Blasts lodged in the umbilical cord, chorionic plate, villi and membranes (Panels 25, H&E × 100). Megakaryoblasts were generally confined to vascular lumina, but they infiltrated their walls (Panel 2 right, H&E × 100). Immunohistochemically, blasts expressed CD61 (Panels 2,4,5 × 100 objectiv). Peri‐villous (maternal) space is spared (Panel 6)

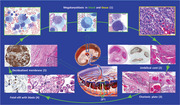

Literature review on placenta involved by TAM show in the majority of cases, peripheral blood leukocytosis exceeded 100 000/μL and blast counts ranged from 23% to 95%. A consistent finding was also the presence of circulating blast cells in the umbilical cord and chorionic villous vasculature. Leukocytosis with left‐shifted hematopoietic elements and myeloid blasts can be seen within vessels of the umbilical cord, chorionic plate, and chorionic villi. Myeloid blasts are generally confined to vascular lumina, but they can infiltrate the vascular wall and stroma to varying degrees. The differential diagnoses are broad. It includes acute villitis, intervillositis, microabscesses caused by listeriosis and *E coli*, and enlarged, hypercellular chorionic villi with lymphoplasmacytic infiltrations, and large inclusion cells in cytomegalovirus infection, all of which can mimic a leukemoid reaction or TAM.

## CONFLICT OF INTEREST

The authors have declared no conflict of interest.

2

## ETHICS

This case followed Loma Linda University and patient ethics.

## Data Availability

The data that support the findings of this study are available from the corresponding author upon reasonable request.

